# Decoding nitrogen depletion–induced lipid accumulation in *Aurantiochytrium* sp. YHPM1 through integrated proteomic and transcriptomic analyses

**DOI:** 10.1186/s12866-026-05058-9

**Published:** 2026-04-21

**Authors:** Shuxian Pang, Hassan Mohamed, Yuanda Song

**Affiliations:** 1 Colin Ratledge Center for Microbial Lipids, School of Agricultural Engineering and Food Science, Shandong University of Technology, Zibo, Shandong 255000 China; 2 Department of Botany and Microbiology, Faculty of Science, Al-Azhar University, Assiut, 71524 Egypt; 3 Shandong Engineering Research Center of Precision Nutrition and Healthy Aging, Qilu Medical University, Zibo, Shandong 255300 China; 4 Research Institute of Natural Products and Health Industry Innovation, Qilu Medical University, Zibo, Shandong 255300 China

**Keywords:** *Aurantiochytrium sp*. YHPM1, Nitrogen depletion, Docosahexaenoic acid (DHA), Post-transcriptional regulation, Lipid accumulation

## Abstract

Docosahexaenoic acid (DHA) is a high-value omega-3 polyunsaturated fatty acid (PUFA) with critical roles in human health and nutrition, and *Aurantiochytrium* species have emerged as promising microbial cell factories for sustainable and scalable DHA biosynthesis, achieving yields of up to 43% of total fatty acids. To elucidate the regulatory mechanisms underlying lipid overproduction in this organism, we performed an integrated proteomic and transcriptomic analysis of the high-yield mutant *Aurantiochytrium* sp. YHPM1 under a two-stage nitrogen depletion strategy, at 48 and 108 h, the strain exhibited a biphasic metabolic response. Initially, β-oxidation and amino-acid catabolism were suppressed. This was followed by enhanced fatty-acid (FA) and triacylglycerol (TAG) biosynthesis. Moreover, key lipid biosynthetic enzymes, including DGAT, DLAT, and GPAT were markedly upregulated at both transcriptomic and proteomic levels, while β-oxidation enzymes, such as ACADM and FOX2 were suppressed. Notably, 8% of transcripts exhibited discordant transcript–protein expression, most prominently at 48 h, suggesting extensive post-transcriptional regulation. Overall, these findings demonstrate a phased metabolic reprogramming strategy in YHPM1 and advance our understanding of post-transcriptional mechanisms governing DHA accumulation, thereby facilitating the industrial-scale production of DHA using *Aurantiochytrium.*

**Supplementary Information:**

The online version contains supplementary material available at 10.1186/s12866-026-05058-9.

## Introduction

Docosahexaenoic acid (DHA; 22:6, n-3) is an essential omega-3 polyunsaturated fatty acid (PUFA) whose global demand has increased markedly in recent years [1]. Dietary DHA has well-established health benefits, including supporting fetal and infant brain and eye development, reducing the risk of preterm labor and cardiovascular disease, and improving cognitive function and visual health in adults and the elderly [2]. Dietary recommendations for PUFAs are provided by various organizations worldwide. For instance, the International Society for the Study of Fatty Acids and Lipids (ISSFAL) recommends a minimum daily intake of 500 mg of DHA to support cardiovascular health [3]. However, current methods for obtaining PUFAs largely rely on extraction from plants and animals or chemical synthesis, which are not sustainable. Consequently, there is an urgent need to develop green and sustainable strategies for PUFA production [4]. Lipid production in oleaginous microorganisms has attracted increasing attention due to its potential applications in nutritional health products. Compared with traditional sources such as fish oil, microbial DHA production offers a scalable and environmentally sustainable alternative, mitigating issues related to overfishing and marine pollution [5].

Thraustochytrids are unicellular heterotrophic marine protozoa belonging to the class Stramenopile, which are often considered to be non-photosynthetic microalgae. These organisms have attracted increasing attention due to their ability to produce high levels of PUFAs, especially DHA. Among this, *Aurantiochytrium* sp. has emerged as a particularly competitive source of DHA, exhibiting higher yields than most other microalgae [1, 6, 7]. Another characteristic is rapid proliferation, and lipids are predominantly deposited in the form of intracellular triacylglycerols (TAGs) [8]. In *Aurantiochytrium*, there are two FA synthesis pathways, the type I FA synthase (FAS) pathway produces saturated FAs, which are mainly composed of palmitic acid (PA, C16:0), the polyketide synthase (PKS) like PUFA synthase pathway involves DHA and docosapentaenoic acid (DPA, C22:5) synthesis [9, 10]. DHA biosynthesis involves multiple enzymatic reactions occurring in different subcellular compartments and requires adequate supplies of acetyl-CoA and NADPH. Therefore, researchers have shown great interest in understanding the molecular mechanisms of DHA production in *Aurantiochytrium*.

Lipid accumulation in oleaginous microorganisms is a complex process governed by tightly coordinated metabolic and regulatory networks. Proteomics offers a powerful tool to explore this complexity by capturing changes in protein abundance, translational activity, and post-translational modifications [11]. In parallel, multi-omics approaches, including genomics, transcriptomics, metabolomics, and proteomics, have been widely applied to elucidate these intricate regulatory systems [12], Among these, the integration of transcriptomics and proteomics is particularly valuable for studying lipid biosynthesis, as transcriptomics reflects mRNA dynamics while proteomics provides direct insights into functional protein-level changes [13]. In this study, the wild-type *Aurantiochytrium* sp. strain SW1 and its high-DHA-producing mutant YHPM1, generated through plasma radiation followed by zeocin and polydatin treatment [14], were subjected to proteomic analysis under two-stage nitrogen depletion. This approach was designed to uncover key regulatory mechanisms associated with enhanced lipid accumulation in YHPM1. Notably, discrepancies between mRNA and protein levels are frequently observed under nutrient stress conditions, highlighting the importance of post-transcriptional regulation in shaping cellular responses [15]. Therefore, focusing on proteomic dynamics, complemented by transcriptomic context, enables a more accurate reconstruction of the metabolic strategies employed by *Aurantiochytrium* during nitrogen depletion.

In this study, we compared the high-DHA mutant YHPM1 with its wild-type parent SW1 at two key time points: 48 h following nitrogen depletion and 108 h at peak DHA accumulation. By integrating proteomic and transcriptomic data, we reconstructed the FA and DHA biosynthesis pathways and elucidated the molecular mechanisms underlying YHPM1’s enhanced lipid production.

## Materials and methods

### Strains and culture conditions

The strains used in this study were *Aurantiochytrium* sp. SW1 (GenBank: KF500513) and *Aurantiochytrium* sp. YHPM1. SW1 was provided by the Laboratory of Microbial Physiology, Faculty of Biological Sciences and Biotechnology, National University of Malaysia, and the Colin Ratledge Microbial Lipid Research Center, Shandong University of Technology. It has been deposited in the UN Microorganism Collection under the accession number UPMC 963. The YHPM1 strain was also supplied by the Colin Ratledge Microbial Lipid Research Center at Shandong University of Technology. Both strains were cultured on diagonal plates of seawater nutrient agar (SNA), which contained 28 g/L of nutrient agar and 17.5 g/L of artificial seawater, at a salinity of 50% (w/w) [14], and incubated at 28 °C for up to 48 h, with an agitation speed of 200 rpm and an initial pH of 6.5. Seed cultures were prepared by inoculating 100 mL of SNA slant agar strips containing 10 colonies of 48 h-old SW1 cells into 500 mL of Erlenmeyer flasks. The culture medium for seed cultivation contained 60 g/L glucose, 2 g/L yeast extract, 10 g/L sodium glutamate, and 6 g/L artificial sea salt. Cultivation was carried out at 28 °C for up to 130 h, with an agitation speed of 600 rpm and an initial pH of 6.5. The production medium contained glucose and sea salt, while the nitrogen sources, monosodium glutamate and yeast extract, were replaced by peptone and tryptone at 8 and 4 g/L, respectively. For proteomic analyses, cultures were grown under nitrogen depletion conditions and sampled at 48 h (hereafter SW48 and YH48 for SW1 and YHPM1, respectively), and 108 h (SW108 and YH108).

### Protein extraction and peptide digestion

Triplicate samples (50 mL) were collected at the selected time points (48 h : nitrogen depletion stage and 108 h : DHA maximizing stage) based on growth and lipid profile analyses, then mixed with an appropriate volume of SDT lysis buffer, boiled for 3 min, sonicated for 2 min, and centrifuged at 16,000 × g for 20 min at 4 °C. The supernatant was collected and protein concentration determined by the BCA assay. For FASP digestion, proteins were reduced with 100 mM DTT (boiled 5 min, then cooled), combined with 200 µL UA buffer (8 M urea, 150 mM Tris-HCl, pH 8.0) and loaded onto 10 kDa ultrafiltration units, with two UA washes (12000 × g, 15 min each). Samples were alkylated with 100 µL 50 mM IAA in UA by vortexing (600 rpm, 1 min) and incubating in the dark at room temperature for 30 min, followed by two more UA washes (12000 × g, 10 min each) and two exchanges into 50 mM NH₄HCO₃ buffer (14000 × g, 10 min each). Digestion was carried out by adding 6 µg trypsin in 40 µL 50 mM NH₄HCO₃, vortexing (600 rpm, 1 min), and incubating at 37 °C for 16–18 h. Peptides were recovered by centrifugation (12000 × g, 10 min), acidified with 0.1% trifluoroacetic acid, desalted on a C18 cartridge, and lyophilized. Finally, the dried peptides were resuspended in 0.1% formic acid, quantified, and prepared for liquid chromatography-mass spectrometry (LC-MS) analysis.

### DIA Mass spectrometry data acquisition

Peptide aliquots from each sample were subjected to chromatographic separation on a Vanquish Neo UHPLC system (Thermo Scientific). The buffers were 0.1% formic acid in water for liquid A and 0.1% formic acid in acetonitrile (80% acetonitrile) for liquid B. The chromatographic separation was carried out on a 96% A column. The column was equilibrated with 96% of liquid A. The sample was injected into the Trap Column. The samples were injected into a Trap Column (PepMap Neo 5 μm C18 300 μm X 5 mm, Thermo Scientific) and passed through a gradient separation on a chromatographic column (µ PAC Neo High Throughput column, Thermo Scientific).

The liquid-phase gradient was set as follows: 0–0.8 min, mobile phase B from 4% to 8%; 0.8–15.1 min, mobile phase B from 8% to 22.5%; 15.1–20.1 min, mobile phase B from 22.5% to 45%; and 20.1–24 min, liquid B maintained at 99%. The peptides were separated and analysed by DIA (data-independent acquisition) mass spectrometry using an Orbitrap Astral mass spectrometer (Thermo Scientific). The analysis time was 24 min, electrospray voltage was 2.2 kV, detection mode: positive ions, the scanning range of parent ions was 380–980 m/z, the resolution of primary mass spectrometry was 240,000, the AGC target was 500%, the Maximum IT of primary mass spectrometry was 3 ms, the resolution of secondary mass spectrometry was 80,000, the AGC target was 500%, the Maximum IT of secondary mass spectrometry was 3 ms, and the RF resolution was 80,000, the AGC target was 500%, the Maximum IT of secondary mass spectrometry was 3 ms. Maximum IT: 3 ms, RF-lens: 40%, MS2 Activation Type: HCD, Isolation window: 2 Th, Normalized collision energy: 25%, cycle time: 0.6.

### Transcriptomic analysis

Total RNA was isolated from biological replicates at each time point with TRIzol reagent (Sangon Biotech, China). RNA integrity was checked before library preparation. Poly(A)+ mRNA was enriched by oligo(dT) magnetic beads, fragmented, and reverse-transcribed into cDNA. Second-strand cDNA synthesis and PCR amplification were performed sequentially. All constructed libraries were qualified with an Agilent 2100 Bioanalyzer, then sequenced via paired-end mode on an Illumina HiSeq platform at Bioprofile Biotechnology Co., Ltd. (Shanghai, China). Raw sequencing reads were filtered to generate clean reads and aligned against reference databases. Gene functional annotation was conducted using Nr, KEGG, and Gene Ontology (GO) databases. Transcript expression levels were calculated based on fragments per kilobase of transcript per million mapped reads (FPKM). Genes with FDR ≤ 0.01 and |log₂FC| ≥ 1 were defined as differentially expressed genes (DEGs). For integrative analysis, transcriptomic data were combined with proteomic data to investigate lipid metabolism and DHA biosynthesis pathways.

### RT-qPCR validate

To validate the reliability of transcriptomic data, several key differentially expressed genes were selected for RT-qPCR analysis. Total RNA was extracted from samples at corresponding time points, reverse-transcribed into cDNA, and subjected to quantitative PCR using gene-specific primers. The relative expression levels were calculated using the 2^-ΔΔCt method. Detailed primer sequences are provided in Supplementary Table S1.

### Data analysis and statistics

Proteomic data were processed using Spectronaut software with a Q-value threshold of ≤ 0.01. Differentially expressed proteins (DEPs) were defined as those with |log₂FC| ≥ 1 and P-value < 0.05. All statistical analyses were performed using SPSS Statistics version 22.0 (IBM, USA), and data are presented as mean ± standard deviation (SD) from at least three biological replicates. Based on KEGG pathway annotation, 392 proteins related to lipid metabolism were selected. Hierarchical clustering analysis was performed using the log₂ transformed fold changes (YHPM1 and SW1) at 48 h and 108 h as input. Missing values were filled using the median value of each protein, and then all proteins were standardized using Z-score. Hierarchical clustering analysis was conducted in R (version 4.2) using the pheatmap package, with Euclidean distance and Ward’s connection method (ward.D2) employed. Clusters were defined based on the protein expression patterns at the two time points, and a row-normalized heatmap was generated to visualize the overall proteome changes. Detailed clustering results are presented in Supplementary Table S1.

## Results

### Global proteomic landscape of SW1 vs. YHPM1

**Fig. 1:**
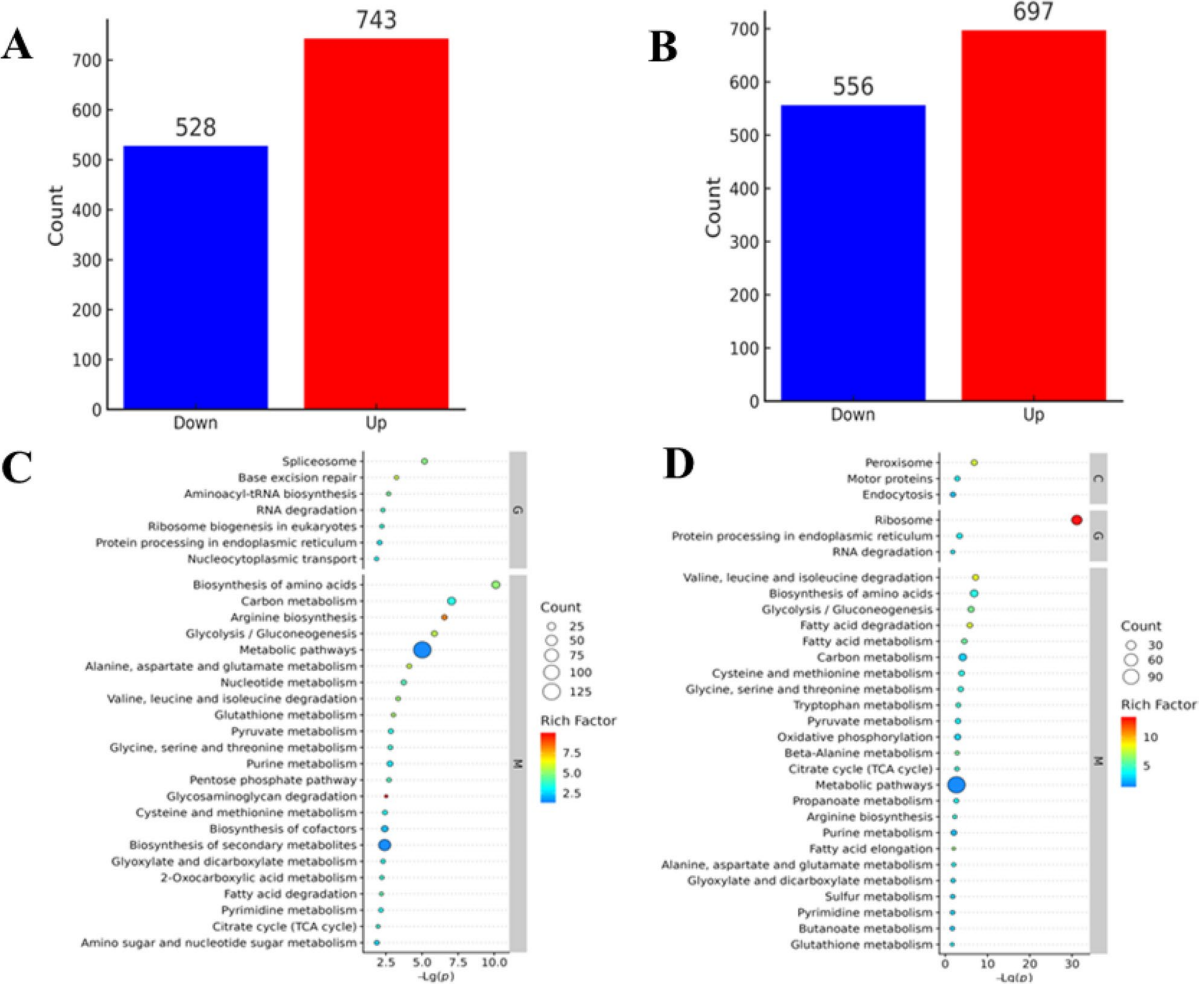
Differential protein profiles and KEGG pathway enrichment under nitrogen depletion. **A** Number of significantly upregulated and downregulated proteins at 48 h in YHPM1 vs. SW1. **B** Number of significantly upregulated and downregulated proteins at 108 h in YHPM1 vs. SW1. **C** KEGG pathway enrichment of DEPs at 48 h. **D** KEGG pathway enrichment of DEPs at 108 h

Comparative proteomic analysis (FC > 2.0, *P* < 0.05) of SW48 vs. YH48 and SW108 vs. YH108 identified 743 upregulated and 543 downregulated proteins at 48 h, and 697 upregulated and 556 downregulated proteins at 108 h (Fig. 1A, B). KEGG pathway analysis revealed that at 48 h differentially expressed proteins (DEPs) were significantly enriched in carbon metabolism, glycolysis, secondary metabolite biosynthesis, FA elongation, and glycerolipid metabolism, whereas at 108 h DEPs shifted to FA metabolism, FA biosynthesis, triglyceride metabolism, ribosomal activity, and oxidative phosphorylation (Fig. 1C, D), suggesting a precise metabolic reprogramming that promotes DHA accumulation.

**Fig. 2:**
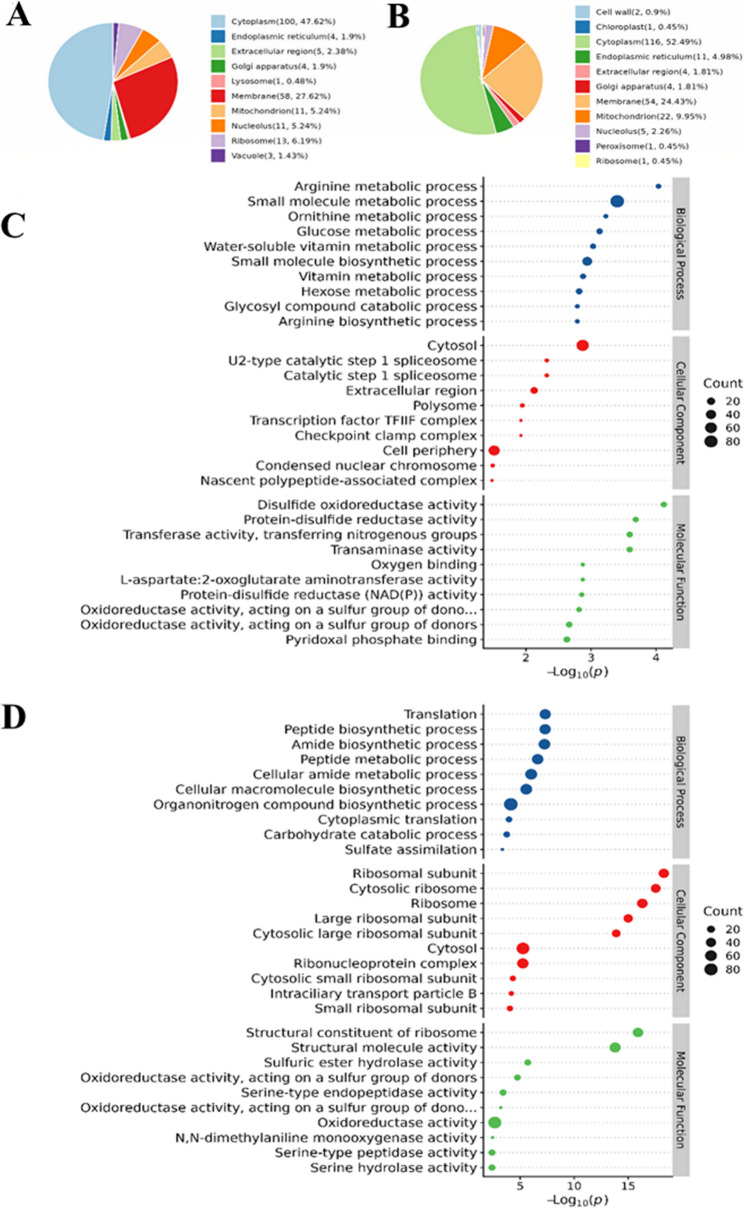
Subcellular localization of DEPs for SW48 vs. YH48 (**A**) and SW108 vs. YH108 (**B**); GO enrichment bubble plots for SW48 vs. YH48 (BP/CC/MF) (**C**) and for SW108 vs. YH108 (**D**). Bubble size indicates protein number in each GO category; color indicates statistical significance (p-value)

To further explore the functional distribution of DEPs, subcellular localization analysis was performed. In the SW48 vs. YH48 comparison, most DEPs were localized in cytoplasm (47.6%), membrane (27.6%), ribosome (6.2%), and mitochondria (5.2%) (Fig. 2A). Similarly, in the comparison of SW108 vs. YH108, DEPs were mainly distributed in cytoplasm (52.5%), membrane (24.4%), mitochondria (9.9%), and endoplasmic reticulum (5.0%) (Fig. 2B). Gene ontology (GO) analysis classified the DEPs into biological process (BP), molecular function (MF) and cellular component (CC). Enrichment analysis showed that at 48 h they were mainly involved in small‑molecule metabolism and biosynthesis with prominent oxidoreductase and ribosomal activities (Fig. 2C), while at 108 h the focus shifted to macromolecule and small‑molecule metabolism with continued oxidoreductase prominence (Fig. 2D). In summary, DEPs at both time points primarily localized to the cytoplasm and membrane. GO enrichment consistently highlighted metabolic and biosynthetic processes, with oxidoreductase activity strongly represented in both stages. These results indicate a largely conserved functional distribution of DEPs over time.

### Dynamics of lipid metabolism and intersecting enzymes

**Fig. 3:**
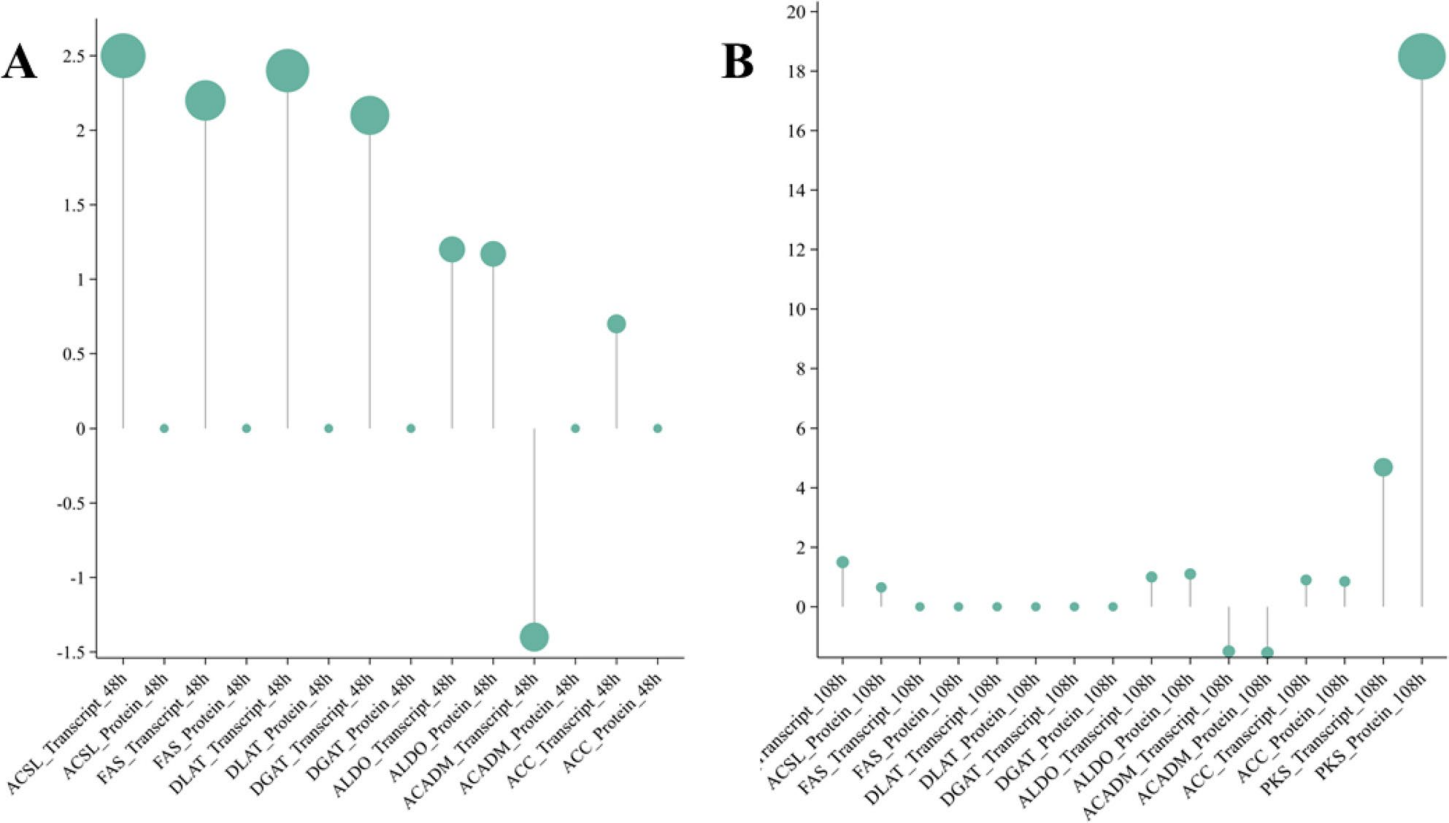
Transcript and protein expression dynamics of key lipid metabolic enzymes in YHPM1 at 48 h (**A**) and 108 h (**B**). Abbreviations: ACSL: Acyl-CoA synthetase long-chain family member; FAS: Fatty acid synthase; DLAT: Dihydrolipoamide acetyltransferase; DGAT: Diacylglycerol O-acyltransferase; ALDO: Fructose-bisphosphate aldolase; ACADM: Acyl-CoA dehydrogenase medium-chain; ACC: Acetyl-CoA carboxylase; PKS: Polyketide synthase

To elucidate the dynamic changes in lipid metabolism during DHA accumulation, DEPs were analyzed at both the nitrogen depletion stage (SW48 vs. YH48) and the DHA-maximizing stage (SW108 vs. YH108), with particular emphasis on enzymes involved in lipid metabolism (Fig. 3). Notably, YHPM1 exhibited a 52.8% increase in DHA content compared to SW1. At 48 h, the mRNA levels of the four synthetic enzymes ACSL (Acyl-CoA synthetase long-chain family member), FAS (Fatty acid synthase), DLAT (Dihydrolipoamide acetyltransferase), and DGAT (Diacylglycerol O-acyltransferase) were significantly upregulated (log₂FC > 2.0), but their protein intensities did not reach the quantification threshold (ND); in contrast, the proteins of ALDO (Fructose-bisphosphate aldolase) and ACADM (Acyl-CoA dehydrogenase medium-chain) were quantifiable, with changes of log₂FC = 1.17 and − 1.55, respectively. These results suggest that lipid metabolism may be transcriptionally activated at an early stage; however, the limited detection of corresponding proteins could reflect post-transcriptional regulation, low protein abundance, rapid turnover, or technical limitations of proteomic detection.

By 108 h, proteins of ACC (Acetyl-CoA carboxylase), ALDO, and ACSL became detectable (log₂FC = 0.85, 1.10, and ~ 0.65, respectively), while ACADM remained ND, supporting the idea that targeted protein synthesis resumed during the lipid accumulation phase. Transcriptomic data showed significant upregulation of PKS-related genes at 108 h (e.g., TRINITY_DN9834_c0_g1.p1, KEGG: K24622, max log₂FC = 4.69), with no such genes detected at 48 h. Notably, the PKS protein TRINITY_DN9834_c0_g1.p1 was identified with marked upregulation in the 108 h proteome, indicating activation of PKS-related pathways at both transcriptional and protein levels during lipid accumulation.

These results indicate that YHPM1 employs adaptive regulatory strategies during early nitrogen depletion. At 48 h, transcripts of key lipid biosynthetic enzymes (e.g., FAS, ACSL, and DGAT) were significantly upregulated, yet their proteins remained undetectable. This discrepancy may reflect potential translational regulation or delayed protein accumulation; however, alternative explanations, such as limitations in proteomic detection sensitivity, cannot be excluded.

### Integrated metabolic pathway and global protein expression changes during lipid accumulation

**Fig. 4:**
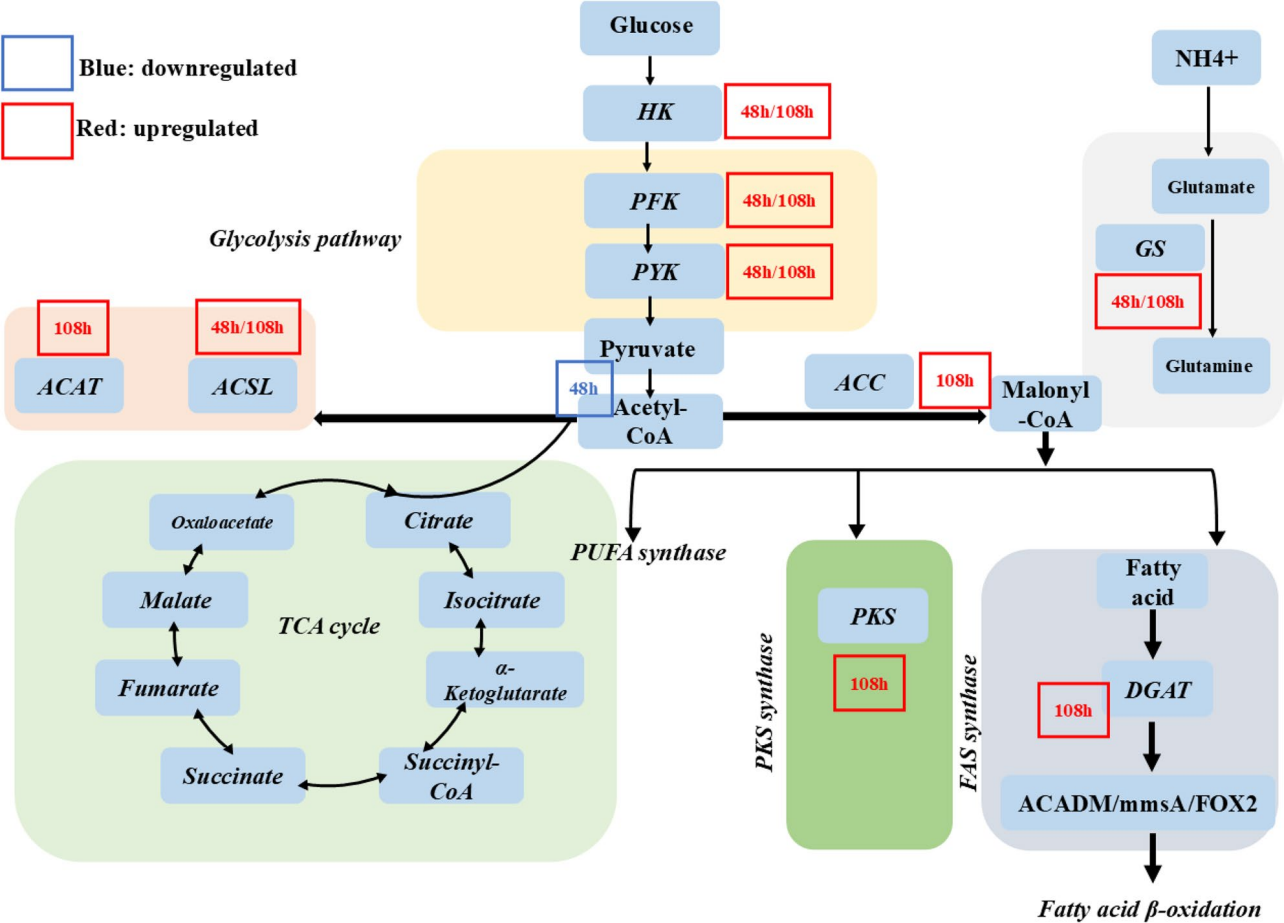
Key metabolic enzyme expression alterations. Different metabolic modules are now distinguished by colored background blocks, clearly illustrating the differences between categories of metabolic pathways. Within each module, metabolites are presented in blue boxes with black regular font; enzymes are shown in blue boxes with black italic font; and metabolic pathway names are displayed in black italic font

To gain a comprehensive understanding of the metabolic changes during DHA accumulation in YHPM1, we integrated the enzyme level expression data into a simplified metabolic pathway map (Fig. 4). Under nitrogen depletion conditions for 48 h, the transcripts of ACSL, FAS, DLAT, and DGAT were significantly upregulated, but their protein levels remained below the detection threshold, while the expression levels of ALDO and ACADM proteins increased moderately (log₂FC values of 1.17 and − 1.55, respectively). By 108 h, proteomics analysis detected 12 out of 15 key biosynthetic enzymes: ACC (protein log₂FC = 0.85), FAS (0.67), ACSL (0.72), and DGAT (0.94) showed a marked increase, consistent with the transcript trend, while catabolic enzymes such as ACADM (-1.10) and FOX2 (-0.95) remained suppressed. The integrated pathway diagram illustrates how cells redirect their metabolism over time under nitrogen depletion. At 48 h, transcriptional upregulation of ACSL, FAS, DLAT, and DGAT indicates robust initiation of the lipid synthesis network, but translation is selectively delayed, potentially preventing premature transfer of acetyl-CoA to ngation. In contrast, by 108 h, most core biosynthetic enzymes (including ACC, FAS, and DGAT) are actively translated, enabling rapid TAG assembly. PKS pathway genes were specifically upregulated at 108 h. By selectively translating key enzymes, the cell directs metabolic flux toward FAS-derived FAs to support TAG accumulation, while PKS pathway proteins remain undetectable, suppressing PUFA synthesis during energetically unfavorable phases. Overall, this comprehensive analysis of metabolic pathways reveals, at the systems level, how metabolic pathways are reorganized during nitrogen depletion. By integrating transcriptomic and proteomic data, the analysis highlights coordinated changes in pathway activity across different stages. Transcriptional upregulation in the early stages may contribute to the initiation of lipid synthesis pathways, while subsequent changes at the protein level are associated with the execution of triglyceride assembly. Notably, this approach identified differential regulatory patterns among key pathways (such as FAS and PKS), suggesting the possibility of post-transcriptional regulation and stage-specific pathway activation.

**Fig. 5:**
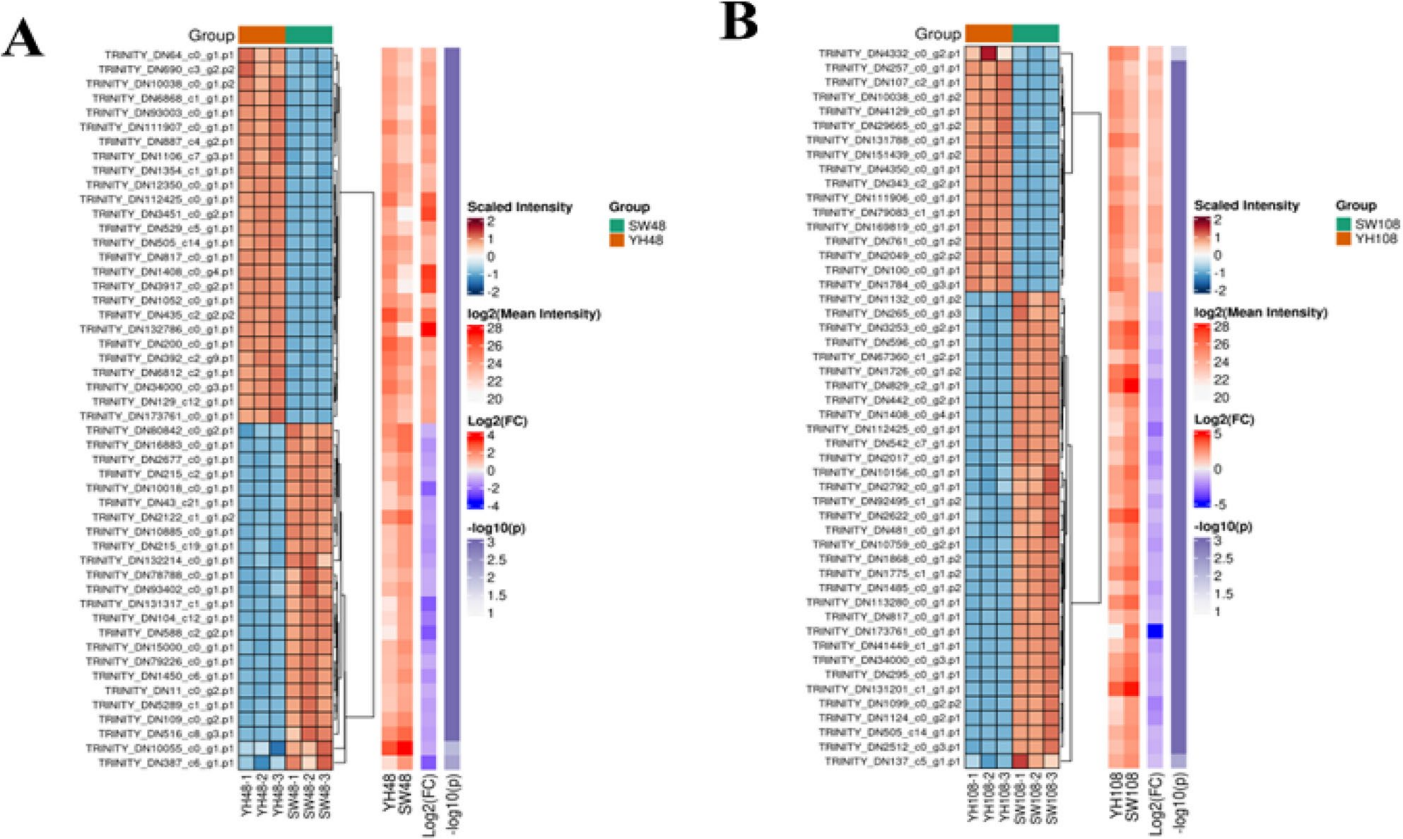
Heat map of differentially expressed proteins at 48 h (**A**) and 108 h (**B**)

We also created a heat map of all lipid metabolism–related proteins to display global proteomic shifts at both time points (Fig. 5). Meanwhile, to quantitatively evaluate the whole genome proteome remodeling, we examined all lipid-associated proteins (*n* = 392; Ward hierarchical clustering analysis was conducted on the log₂FC values as shown in Supplementary Table S2). Clustering divides them into two main groups:

Cluster 0 (*n* = 334) : Proteins that were significantly upregulated at 108 h were enriched in biosynthetic machinery (such as ACC, FAS, ACSL, DGAT), reflecting the selective activation of lipid synthesis modules. Cluster 1 (*n* = 58) : Proteins that were continuously downregulated at 48 h and 108 h, mainly composed of β-oxidase (such as ACADM, FOX2), indicating that the degradation pathway was continuously inhibited. The clustering pattern highlights a global shift from catabolic inhibition to anabolic activation. Proteins in Cluster 0 were predominantly upregulated at 108 h, representing biosynthetic mechanisms required for FA elongation, acyl-CoA activation, and TAG assembly, reflecting the late-stage metabolic commitment to lipid accumulation. Conversely, Cluster 1 contains core enzymes for β-oxidation and lipid conversion, whose sustained downregulation confirms prolonged inhibition of FA degradation. Collectively, these clusters illustrate how YHPM1 reprograms its proteome to minimize carbon loss while maximizing storage lipid synthesis under prolonged nitrogen stress.

This holistic view revealed a significant shift from catabolism to anabolism, highlighting the coordinated reprogramming of metabolic pathways at the proteomic level. This comprehensive visualization clearly demonstrated how YHPM1 strategically regulated metabolic flux under nitrogen depletion to balance energy conservation and lipid biosynthesis.

### Transcript–protein correlation analysis reveals post-transcriptional modulation

**Fig. 6:**
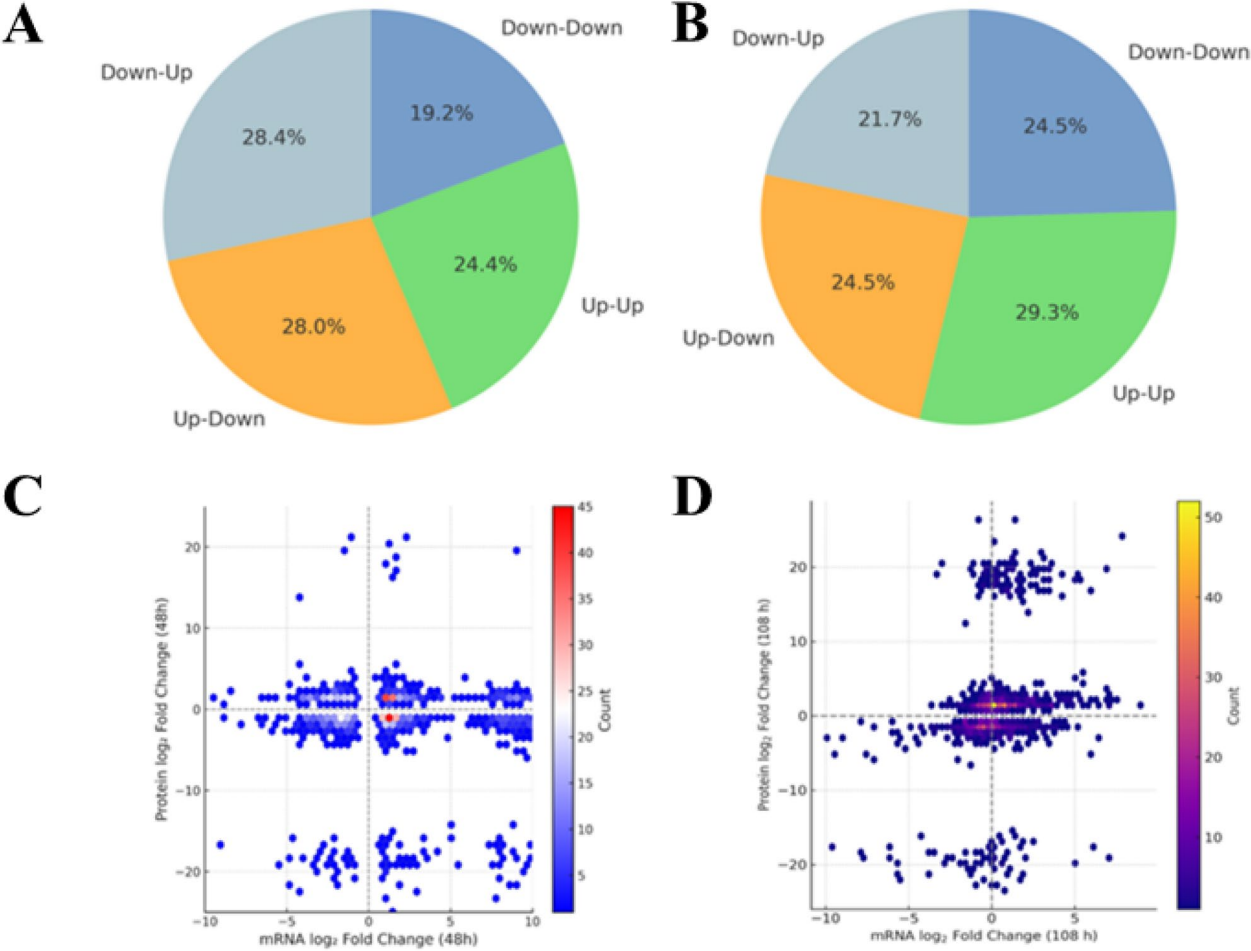
48 h and 108 h transcript-protein correlations and co-regulation. **A** Distribution of mRNA/protein co-regulatory categories at 48 h (Up-Up, Up-Down, Down-Up, Down-Down). **B** Distribution of mRNA/protein co-regulatory categories at 108 h. **C** Scatter plot of log-fold changes in mRNA vs. protein at 48 h. **D** Scatter plot of log 2-fold changes in mRNA versus protein at 108 h

We matched 9,838 gene-protein pairs to compare the fold change of mRNA and protein abundance at 48 h and 108 h. Overall, transcript and protein levels showed a moderate positive correlation (Pearson’s *r* = 0.67), a weak negative correlation at 48 h (*r* = -0.21), and a moderate positive correlation at 108 h (*r* = 0.65) (Fig. 6A-B). Focusing on 312 lipid metabolism–related pairs, 42% were Up–Up, 33% Down–Down, 15% Up–Down, and 10% Down–Up (Fig. 6C- D), indicating that 75% of the transcriptional and protein regulation were consistent and 25% were inconsistent. These results indicate that post-transcriptional regulation plays a dominant role in the early nitrogen depletion stage, while during the peak period of DHA accumulation, the transcription-translation coupling is tighter. The significant temporal decoupling between mRNA and protein expression at 48 h (*r* = -0.21) strongly indicates substantial post-transcriptional regulatory mechanisms controlling the initiation of lipid synthesis pathways.

## Discussion

*Aurantiochytrium* sp. has recently attracted considerable attention as an efficient microbial source of PUFAs, particularly DHA [1]. DHA is an essential structural component of cellular membranes and plays critical roles in neuronal development, retinal function, and immune regulation, as well as the prevention of neurodegenerative and cardiovascular disease [16]. Consequently, enhancing microbial DHA production and elucidating its underlying regulatory mechanisms are of significant biological and industrial importance. Nitrogen depletion is widely recognized as an effective strategy to induce lipid accumulation in oleaginous microorganisms [17], however, the detailed metabolic strategies and regulatory mechanisms governing this process remain incompletely understood. In the present study, we identified a distinct two-stage metabolic reprogramming pattern in the high-DHA-producing mutant strain *Aurantiochytrium* sp. YHPM1. This pattern is characterized by dynamic shifts between metabolic inhibition and activation at both transcriptional and post-transcriptional levels during nitrogen depletion. While similar lipid accumulation strategies have been reported in other microalgae, such as *Chlorella* and *Neochloris oleoabundans* [18–20], the regulatory dynamics observed in YHPM1 appear to follow a different pattern. This distinction highlights the unique metabolic adaptability of *Aurantiochytrium* and underscores its potential as a promising platform for sustainable DHA production. Notably, these multi-layered regulatory events suggest that post-transcriptional control is not merely supplementary but constitutes a core regulatory strategy governing metabolic reprogramming under nitrogen limitation [21].

Under nitrogen depletion (48 h), YHPM1 exhibited a marked suppression of key catabolic pathways, particularly fatty acid (FA) β-oxidation and amino acid degradation, which likely contributed to the preservation of carbon skeletons and a reduction in energy expenditure associated with unnecessary turnover processes [22, 23]. Notably, the consistent downregulation of ACADM and FOX2 (log₂FC = − 1.55 and − 0.95 at 48 h and 108 h, respectively) suggests a reduced capacity for lipid degradation, thereby favoring the retention of intracellular carbon for lipid and DHA biosynthesis [24]. During the later DHA maximization phase (108 h), a coordinated upregulation of core lipid biosynthetic enzymes, including ACC, FAS, ACSL, and DGAT, was observed at both transcriptomic and proteomic levels, supporting enhanced FA elongation, desaturation, and TAG assembly [25]. A similar trend has been reported in *Aurantiochytrium* sp. T66, where elevated FAS expression under nitrogen depletion reflects increased lipid biosynthetic capacity [17]. Consistently, proteomic data in this study indicated increased abundance of key metabolic intermediates and enzymes associated with glycerol-3-phosphate, acetyl-CoA, and TAG biosynthesis, further supporting the activation of lipid-producing pathways in YHPM1.

The observed two-stage metabolic reprogramming highlights tight regulation of carbon flux under nitrogen-limited conditions. In oleaginous yeasts such as *Yarrowia lipolytica*, intracellular fatty acids are primarily degraded via peroxisomal β-oxidation or ω-oxidation pathways [26]. Suppression of these pathways, as observed in YHPM1, may contribute to intracellular accumulation of acetyl-CoA, a central metabolic intermediate linking glycolysis, the tricarboxylic acid cycle, and FA biosynthesis [27, 28]. By limiting acetyl-CoA consumption through catabolic routes while enhancing its utilization in anabolic lipid pathways, YHPM1 appears to redirect carbon flux toward TAG and DHA accumulation [29]. In addition, the biosynthesis of long-chain PUFAs such as DHA requires substantial amounts of ATP and NADPH. This demand may be supported by a metabolic shift favoring glycolysis over gluconeogenesis under nitrogen depletion conditions [30, 31]. Such coordinated regulation further emphasizes that improving acetyl-CoA availability and its efficient channeling into lipid biosynthesis is a key strategy for enhancing lipid production in oleaginous microorganisms [32]. Interestingly, although ACC expression was slightly downregulated in YHPM1, a similar trend has been reported in *Phaeodactylum tricornutum*, where ACC (B7G7S4) showed only minor downregulation under nitrogen depletion (log₂FC = − 1.02; *p* = 0.70) [32]. This observation supports the notion that lipid accumulation may rely more on the redistribution of metabolic flux toward acetyl-CoA utilization rather than on increased ACC expression alone.

Upregulation of central carbon metabolism, including glycolysis and the early (48 h) TCA cycle, provides key precursors such as acetyl-CoA and reducing equivalents (e.g., NADPH) for lipid biosynthesis [33]. Protein subcellular localization provides important clues for interpreting functional proteomic changes, particularly those related to transcriptional activity and mitochondrial energy metabolism [34, 35]. In summary, proteomic profiling indicates that YHPM1 undergoes tightly coordinated metabolic shifts under nitrogen depletion [1]. At 48 h, enrichment of glycolysis, carbon metabolism, and ribosomal pathways suggests a focus on carbon acquisition and energy balance while limiting energy-intensive biosynthesis [36]. In contrast, at 108 h, enhanced fatty acid biosynthesis and oxidative phosphorylation reflect a transition toward lipid accumulation and sustained energy production [25]. Overall, these changes highlight a phased metabolic strategy in which YHPM1 initially reallocates carbon resources before channeling them into intensive lipid and DHA synthesis.

Concurrently, enzymes such as ALDO remain actively translated to sustain glycolytic flux and ensure continuous substrate supply [37]. whereas β-oxidation enzymes (e.g., ACADM) remain suppressed, minimizing unnecessary fatty acid turnover [38, 39]. Collectively, YHPM1 adopts a “prepare first, produce later” strategy, where mRNA is initiated early but translation is delayed until lipid assembly becomes metabolically advantageous. This difference between transcription and translation is not random, but likely reflects a regulatory process, allowing cells to accumulate mRNA first and delay protein synthesis to save energy [40]. At 48 h, the observed negative correlation between mRNA and protein levels may reflect multi-layered post-transcriptional regulation, such as selective ribosome recruitment and inhibition of translation initiation [41]. Under nitrogen deficiency, cells can maintain mRNA in a translationally silenced state until conditions improve, thereby rapidly producing proteins without requiring new transcription [42, 43]. This may help cells respond quickly at the transcription level while saving energy, suggesting that post-transcriptional regulation plays an important role under stress conditions [21]. By 108 h, the positive correlation indicates this translational repression is released, making mRNA levels a more reliable predictor of protein abundance. This shift from decoupled to coupled regulation illustrates how YHPM1 dynamically reallocates its metabolic resources under nitrogen stress. This indicates that both transcription and translation play a role in controlling carbon flow toward lipid synthesis [44]. The increase in glycolytic enzyme expression and the inhibition of β-oxidation support the hypothesis that YHPM1 prioritizes the carbon flux of glucose towards FA synthesis rather than energy dissipation. This is consistent with previous reports that emphasized the importance of expanding the acetyl-CoA library in promoting high lipid productivity in oil-bearing microorganisms [32, 45]. Secondly, the triglyceride metabolic pathway in YHPM1 was also activated, especially at 108 h, as confirmed by the upregulation of DGAT and GPAT, which are responsible for the assembly of TAG [13, 46]. The enhanced expression of these enzymes indicates that TAG biosynthesis is not only induced transcriptionally but also supported by proteomics in the mutant. The effective guidance of fatty acyl-coenzyme A and glycerol-3-phosphate in TAG biosynthesis may be the reason for the increased DHA content observed in YHPM1 [13]. This metabolic strategy bears resemblance to patterns reported in oleaginous algae such as *Chlorella* sp. and *Neochloris oleoabundans*, where lipid accumulation involves early inhibition of degradation and late induction of synthesis pathways [18, 47].

Proteomic responses to nitrogen depletion vary widely among microalgae [48], For example, *Thalassiosira pseudonana* shows little change in proteins related to FA and TAG biosynthesis under nitrogen stress [49], in contrast to the patterns observed in this study. Acetyl-CoA carboxylase (ACC), a key enzyme supplying malonyl-CoA for both FAS and PKS pathways [20, 46]. Consistent with this, our proteomic data show that the ACC protein (log₂FC = 0.85) significantly increased after 108 h, indicating an enhanced availability of precursors for downstream lipid and DHA biosynthesis. Surprisingly, although multiple PKS genes showed significant transcriptional level induction in the early stage (48 h) and the late stage (108 h), these PKS proteins were still undetectable. This obvious disconnection highlights the potential translational or post-translational control mechanisms that selectively inhibit the accumulation of PKS proteins while allowing for the efficient translation of ACC and FAS proteins to promote lipid accumulation under nitrogen-deficient conditions [29, 50, 51]. This suggests that key biosynthetic pathways are regulated differentially at multiple levels, with the aim of prioritizing metabolic efficiency rather than uniformly increasing all lipid-related proteins [21].

To better explain the transcription-protein decoupling phenomenon observed in YHPM1 under nitrogen-deficient conditions, we referred to a conceptual framework proposed by Venkataramanan et al., which outlines three possible scenarios explaining the mismatch between mRNA and protein levels under stress conditions [52]. The PKS pathway likely belongs to Scenario II, as its genes are transcriptionally upregulated while the corresponding proteins remain undetectable. This supports that the difference between mRNA and protein levels is not random, but related to how cells respond under stress conditions.

In our study, the first scenario refers to a subset of mRNAs that remain poorly translated under both stress and non-stress conditions. Such sustained inhibition may arise from intrinsic mRNA structural features or reliance on small RNAs for translation initiation. However, our data did not reveal transcripts that consistently exhibited high mRNA levels coupled with low protein abundance across all conditions, suggesting that this mechanism is not a dominant feature in YHPM1. The second scenario involves mRNAs that are only selectively inhibited under stress conditions. In this case, the translation process will be actively inhibited by environmental signals (such as nitrogen limitation). This mechanism is consistent with our observation at 48 h, when genes including ACSL, DGAT and FAS showed significantly increased transcription levels, but their proteins were not detected or remained unchanged. These findings suggest that YHPM1 may temporarily inhibit the translation of energy-intensive enzymes in the early stress response, possibly delay lipid synthesis until the carbon precursor is fully accumulated [44, 53].

The third scenario focuses on proteins accumulated under stress, although their transcription levels are stable or low. This may be achieved by enhanced translation efficiency or increased protein stability. In our study, this pattern was observed in genes such as ALDO and ACADM, where protein abundance increased moderately at 48 h, while the corresponding mRNA levels remained basically unchanged [21]. Additionally, only Δ-8 desaturase was detected at the protein level in YHPM1, whereas other desaturases (e.g., Δ-4, Δ-6, and Δ-9) identified in SW1 were not detected. This selective expression pattern may indicate post-transcriptional regulation, differential protein stability, or strain-specific enzymatic dynamics.

Elucidation of these key regulatory nodes and metabolic strategies provides valuable targets for the rational engineering of *Aurantiochytrium* sp. YHPM1 and other oleaginous microorganisms. In light of the growing global demand for sustainable DHA, these findings strengthen the potential of *Aurantiochytrium* as a platform for large-scale industrial production. Future research should focus on precise genetic manipulation of critical metabolic nodes to further improve production efficiency, thereby enhancing the economic viability and environmental sustainability of microbial DHA biosynthesis as an alternative to traditional marine sources.

The regulatory features identified in this study provide several promising targets for strain improvement. Enhancing the activity of key biosynthetic enzymes, such as DGAT and DLAT, may further increase TAG assembly efficiency, while fine-tuning the expression of ACC, ACSL, and FAS could optimize the supply of acetyl-CoA and acyl-CoA precursors [54]. Additionally, the sustained suppression of β-oxidation enzymes (e.g., ACADM and FOX2) suggests that further limiting FA degradation may help improve carbon retention and support higher lipid accumulation [25]. Furthermore, the strong transcriptional induction of PKS genes, coupled with limited protein-level detection, suggests that this pathway may represent a potential bottleneck in DHA biosynthesis [55, 56]. Enhancing PKS protein stability or improving translational efficiency could help alleviate this constraint and increase PUFA yields [57]. Collectively, these findings provide practical targets for the rational engineering of *Aurantiochytrium* strains to achieve more efficient and economically viable DHA production at an industrial scale [58]. Overall, our findings emphasize that metabolic reprogramming in YHPM1 is governed not only at the transcriptional level but also through dynamic and condition-specific post-transcriptional regulation, which plays a decisive role in coordinating carbon flux and lipid accumulation under nitrogen limitation.

## Conclusions

Our findings provide a comprehensive model of staged metabolic reprogramming in *Aurantiochytrium* sp. YHPM1, driven by both transcriptional and post-transcriptional regulation. This layered control enables the strain to suppress unnecessary energy expenditure while optimizing carbon flow toward DHA rich TAG accumulation. By elucidating key regulatory nodes, such as the early inhibition of β-oxidation, post-transcriptional control of enzyme expression, and the central role of DGAT and DLAT. These insights provide critical genetic targets for future metabolic engineering, offering a sustainable and economically viable alternative to traditional DHA sources such as fish oil.

## Supplementary Information

Supplementary Material 1.

Supplementary Material 2.

## Data Availability

All data generated and/or analyzed during this study are included in this published article and the additional information file.

## References

[1] Prabhakaran P, Nazir MYM, Thananusak R, Hamid AA, Vongsangnak W, Song Y. Uncovering global lipid accumulation routes towards docosahexaenoic acid (DHA) production in Aurantiochytrium sp. SW1 using integrative proteomic analysis. Biochim et Biophys Acta (BBA) Mol Cell Biology Lipids. 2023;1868(11). 10.1016/j.bbalip.2023.159381. 10.1016/j.bbalip.2023.159381 37625782

[2] Li J, Pora BLR, Dong K, Hasjim J. Health benefits of docosahexaenoic acid and its bioavailability: A review. Food Sci Nutr. 2021;9(9):5229–43. 10.1002/fsn3.2299. 34532031 10.1002/fsn3.2299 PMC8441440

[3] Kris-Etherton PM, Grieger JA, Etherton TD. Dietary reference intakes for DHA and EPA. Prostaglandins Leukot Essent Fat Acids. 2009;81(2–3):99–104. 10.1016/j.plefa.2009.05.011. 10.1016/j.plefa.2009.05.011 19525100

[4] Yan CX, Zhang Y, Yang WQ, Ma W, Sun XM, Huang H. Universal and unique strategies for the production of polyunsaturated fatty acids in industrial oleaginous microorganisms. Biotechnol Adv. 2024;70:108298. 10.1016/j.biotechadv.2023.108298. 38048920 10.1016/j.biotechadv.2023.108298

[5] Xu X, Huang C, Xu Z, Xu H, Wang Z, Yu X. The strategies to reduce cost and improve productivity in DHA production by Aurantiochytrium sp.: from biochemical to genetic respects. Appl Microbiol Biotechnol. 2020;104(22):9433–47. 10.1007/s00253-020-10927-y. 32978687 10.1007/s00253-020-10927-y

[6] Ma Z, Tian M, Tan Y, Cui G, Feng Y, Cui Q, Song X. Response mechanism of the docosahexaenoic acid producer *Aurantiochytrium* under cold stress. Algal Res. 2017;25:191–9. 10.1016/j.algal.2017.05.021.

[7] Ward OP, Singh A. Omega-3/6 fatty acids: Alternative sources of production. Process Biochem. 2005;40(12):3627–52. 10.1016/j.procbio.2005.02.020.

[8] Yang J, Song X, Wang L, Cui Q. Comprehensive analysis of metabolic alterations in Schizochytrium sp. strains with different DHA content. J Chromatogr B-analytical Technol Biomedical Life Sci. 2020;1160:122193. 10.1016/j.jchromb.2020.122193. 10.1016/j.jchromb.2020.122193 32949924

[9] Ma Z, Tan Y, Cui G, Feng Y, Cui Q, Song X. Transcriptome and gene expression analysis of DHA producer *Aurantiochytrium* under low temperature conditions. Sci Rep. 2015;5:14446. 10.1038/srep14446. 26403200 10.1038/srep14446 PMC4585886

[10] Matsuda T, Sakaguchi K, Hamaguchi R, Kobayashi T, Abe E, Hama Y, Ito M. Analysis of Delta12-fatty acid desaturase function revealed that two distinct pathways are active for the synthesis of PUFAs in T. aureum ATCC 34304. pids. J Lipid Res. 2012;53(6):1210–22. 10.1194/jlr.M024935. 22368282 10.1194/jlr.M024935 PMC3351828

[11] Tang X, Chen H, Gu Z, Zhang H, Chen YQ, Song Y, Chen W. Comparative proteome analysis between high lipid-producing strain *Mucor circinelloides* WJ11 and low lipid-producing strain CBS 277.49. J Agricultural Food Chem. 2017;65(24):5074–82. 10.1021/acs.jafc.7b00935. 10.1021/acs.jafc.7b00935 28557429

[12] Huang YZ, Qiu Y, Zhang L, Kuang Q, Luo W. Combined analysis of metabolomic, transcriptomic and proteomics to reveal the underlying mechanism of mucilage disappearance in Brasenia schreberi. Ind Crops Prod. 2025;232(15). 10.1016/j.indcrop.2025.121178.

[13] Jia K, Zheng J, Hu S, Lei D, Jiang M, Zhang J, Cui M, Wang J, Cui Y, Wang F. Comprehensive transcriptomic and proteomic analysis reveals biological changes and potential regulatory mechanisms of endothelial cells under heat stress conditions. J Therm Biol. 2025;130:104138. 10.1016/j.jtherbio.2025.104138. 40408820 10.1016/j.jtherbio.2025.104138

[14] Nazir Y, Phabakaran P, Halim H, Mohamed H, Naz T, Hamid A, A., Song Y. Strategic development of *Aurantiochytrium* sp. mutants with superior oxidative stress tolerance and glucose-6-phosphate dehydrogenase activity for enhanced DHA production through plasma mutagenesis coupled with chemical screening. Front Nutr. 2022;9:876649. 10.3389/fnut.2022.876649. 35558745 10.3389/fnut.2022.876649 PMC9087853

[15] Irshad IU, Sharma AK. Understanding the regulation of protein synthesis under stress conditions. Biophys J. 2024;123(20):3627–39. 10.1016/j.bpj.2024.09.014. 39277792 10.1016/j.bpj.2024.09.014 PMC11494521

[16] Feng Y, Zhu Y, Wang B, Zhao F, Zhang Y, Zhu X, Li H, Yu L. Enhanced docosahexaenoic acid production of *Schizochytrium* sp. H016 utilizing rapeseed meal hydrolysate. Process Biochem. 2026;106:102–10. 10.1016/j.procbio.2025.10.011.

[17] Heggeset TMB, Ertesvag H, Liu B, Ellingsen TE, Vadstein O, Aasen IM. Lipid and DHA-production in *Aurantiochytrium* sp. - Responses to nitrogen starvation and oxygen limitation revealed by analyses of production kinetics and global transcriptomes. Sci Rep. 2019;9(1). 10.1038/s41598-019-55902-4. Article 19470. 10.1038/s41598-019-55902-4 PMC6923395 31857635

[18] Rai V, Muthuraj M, Gandhi MN, Das D, Srivastava S. Real-time iTRAQ-based proteome profiling revealed the central metabolism involved in nitrogen starvation induced lipid accumulation in microalgae. Sci Rep. 2017;7:45732. 10.1038/srep45732. 28378827 10.1038/srep45732 PMC5381106

[19] Rismani-Yazdi BZ, Haznedaroglu C, Hsin, Peccia J. Transcriptomic analysis of the oleaginous microalga Neochloris oleoabundans reveals metabolic insights into triacylglyceride accumulation. Biotechnol Biofuels Bioprod. 2012;5(1):74–80. 10.1186/1754-6834-5-74. 10.1186/1754-6834-5-74 PMC3549901 23006831

[20] Wang Q, Jin W, Zhou X, Chen C, Han W, Mahlia TMI, Wang Q. Enhancing docosahexaenoic acid production in Aurantiochytrium species using atmospheric and room temperature plasma mutagenesis and comprehensive multi-omics analysis. Sci Total Environ. 2024;912:169217. 10.1016/j.scitotenv.2023.169217. 38081429 10.1016/j.scitotenv.2023.169217

[21] Vogel C, Marcotte EM. Insights into the regulation of protein abundance from proteomic and transcriptomic analyses. Nat Rev Genet. 2012;13(4):227–32. 10.1038/nrg3185. 22411467 10.1038/nrg3185 PMC3654667

[22] Ratledge C, Wynn JP. The biochemistry and molecular biology of lipid accumulation in oleaginous microorganisms. Adv Appl Microbiol. 2002;51:1–51. 10.1016/S00652164(02)51000-5. 12236054 10.1016/s0065-2164(02)51000-5

[23] Udayantha HMV, Kim SH, Chen Y, Long J, S. D. N. K. B., Kyung-Il P. Enhancing lipid accumulation in *Tetraselmis* sp.: integrating nitrogen deprivation and glucose supplementation for biofuel production. Biotechnol Biofuels Bioprod. 2025;18:57. 10.1186/s13068-025-02654-1. 40457378 10.1186/s13068-025-02654-1 PMC12128517

[24] Patel A, Karageorgou D, Rova E, Katapodis P, Rova U, Christakopoulos P, Matsakas L. An overview of potential oleaginous microorganisms and their role in biodiesel and omega-3 fatty acid-based industries. Microorganisms. 2020;8(3). 10.3390/microorganisms8030434. 10.3390/microorganisms8030434 PMC7143722 32204542

[25] Chen X, He Y, Liu L, Zhu X, Sen B, Wang G. Nitrogen starvation enhances the production of saturated and unsaturated fatty acids in *Aurantiochytrium* sp. PKU#SW8 by regulating key biosynthetic genes. Mar Drugs. 2022;20(10). 10.3390/md20100621. Article 621. 10.3390/md20100621 PMC9605394 36286445

[26] Liu H, Song Y, Fan X, Wang C, Lu X, Tian Y. *Yarrowia lipolytica* as an oleaginous platform for the production of value-added fatty acid-based bioproducts. Front Microbiol. 2020;11:608662. 10.3389/fmicb.2020.608662. 33469452 10.3389/fmicb.2020.608662 PMC7813756

[27] Li X, Dong Y, Chen K, Perumal AB, Zhan Z, Gouda M, He Y. 13 C-metabolic flux analysis of lipid accumulation in the green microalgae Tetradesmus obliquus under nitrogen deficiency stress. Bioresour Technol. 2023;388:129740. 10.1016/j.biortech.2023.129740. 37717702 10.1016/j.biortech.2023.129740

[28] Li J, Wang W, Li B, Xue Y, Wang X, Liu S, Hu S, Tang J, Yan B, Li T, Xue J. NADP^+^-dependent isocitrate dehydrogenase as a novel target for altering carbon flux to lipid accumulation and enhancing antioxidant capacity in Tetradesmus obliquus. Bioresour Technol. 2024;395., Article 130365. 10.1016/j.biortech.2024.130365. 10.1016/j.biortech.2024.130365 38266784

[29] Vijayan J, Sophie A, Wyatt MJ, Amanda M, Nishikant M W., Wayne R. Nitrogen starvation leads to TOR kinase-mediated downregulation of fatty acid synthesis in the algae Chlorella sorokiniana and Chlamydomonas reinhardtii. BMC Plant Biol. 2024;24(1):753–61. 10.1186/s12870-024-05408-7. 39107711 10.1186/s12870-024-05408-7 PMC11302099

[30] Tian L, Chi G, Lin S, Ling X, He N. Marine microorganisms: natural factories for polyunsaturated fatty acid production. Blue Biotechnol. 2024;1. 10.1186/s44315-024-00012-8. Article 15.

[31] Yin F, Sun X, Luo X, Zheng W, Yin L, Zhang Y, Fu Y. A review on marine microbial docosahexaenoic acid production through circular economy, fermentation engineering, and antioxidant technology. Mar Drugs. 2025;23(6):Artilce256. 10.3390/md23060256. 10.3390/md23060256 PMC12194448 40559665

[32] Longworth J, Wu D, Huete-Ortega M, Wright PC, Vaidyanathan S. Proteome response of Phaeodactylum tricornutum, during lipid accumulation induced by nitrogen depletion. Algal Research-Biomass Biofuels Bioprod. 2016;18:213–24. 10.1016/j.algal.2016.06.015. 10.1016/j.algal.2016.06.015 PMC5070409 27812494

[33] Zhang Y, Wu X, Guo X, Li K, Lu Y, Lin X, Ling X. Functions of aldolase in lipid synthesis of Schizochytrium sp. by gene disruption to switch carbon metabolism. Blue Biotechnol. 2024;1:Article17. 10.1186/s44315-024-00014-6.

[34] Sun LP, Ouyang LL, Bao H, Liu JG, Sun Z, Zhou ZG. (2021a). Comparison between two isoforms of glycerol-3-phosphate acyltransferase in microalga: Subcellular localization and role in triacylglycerol synthesis. *Algal Research, 54*, Artilce 102172. 10.1016/j.algal.2020.102172

[35] Wu C, Zhang H, Yang N, Wang C, Zhang M, Liu N, Lei H. Transcriptomics and proteomics analyses reveal the molecular mechanisms of yeast cells regulated by Phe-Cys against ethanol-oxidation cross-stress. Food Chemritry. 2025;464(Pt 2):141694. 10.1016/j.foodchem.2024.141694. 10.1016/j.foodchem.2024.141694 39442214

[36] Zhang M, Gao Y, Yu C, Wang J, Weng K, Li Q, He Y, Guo Z, Zhang H, Huang J, Li L. Transcriptome analysis of malate-induced *Schizochytrium* sp. FJU-512 reveals a novel pathway for biosynthesis of docosahexaenoic acid with enhanced expression of genes responsible for acetyl-CoA and NADPH accumulation. Front Microbiol. 2022;13., Article 1006138. 10.3389/fmicb.2022.1006138. 10.3389/fmicb.2022.1006138 PMC9589357 36299719

[37] Zeng L, Bi Y, Guo P, Bi Y, Wang T, Dong L, Wang F, Chen L, Zhang W. Metabolic Analysis of Schizochytrium Mutants With High DHA Content Achieved With ARTP Mutagenesis Combined With Iodoacetic Acid and Dehydroepiandrosterone Screening. Front Bioeng Biotechnol. 2021;9., Article 738052. 10.3389/fbioe.2021.738052. 10.3389/fbioe.2021.738052 PMC8637758 34869256

[38] Gao B, Wang F, Huang L, Liu H, Zhong Y, Zhang C. Biomass, lipid accumulation kinetics, and the transcriptome of heterotrophic oleaginous microalga Tetradesmus bernardii under different carbon and nitrogen sources. Biotechnol Biofuels. 2021;14(1). 10.1186/s13068-020-01868-9. 10.1186/s13068-020-01868-9 PMC7789750 33407769

[39] Zhu Z, Sun J, Fa Y, Liu X, Lindblad P. Enhancing microalgal lipid accumulation for biofuel production. Front Microbiol. 2022a;13., Article 1024441. 10.3389/fmicb.2022.1024441. 10.3389/fmicb.2022.1024441 PMC9588965 36299727

[40] Jovanovic M, Rooney MS, Mertins P, Przybylski D, Chevrier N, Satija R, Regev A. Immunogenetics. Dynamic profiling of the protein life cycle in response to pathogens. Science. 2015;347(6226):1259038. 10.1126/science.1259038. 25745177 10.1126/science.1259038 PMC4506746

[41] Dever TE, Green R. The elongation, termination, and recycling phases of translation in eukaryotes. Cold Spring Harb Perspect Biol. 2012;4(7). 10.1101/cshperspect.a013706. Article a013706. 10.1101/cshperspect.a013706 PMC3385960 22751155

[42] De Abreu S, Penalva R, Marcotte LO, E. M., Vogel C. Global signatures of protein and mRNA expression levels. Mol Biosyst. 2009;5(12):1512–26. 10.1039/b908315d. 20023718 10.1039/b908315d PMC4089977

[43] Faghihi MA, Wahlestedt C. Regulatory roles of natural antisense transcripts. Nat Rev Mol Cell Biol. 2009;10(9):637–43. 10.1038/nrm2738. 19638999 10.1038/nrm2738 PMC2850559

[44] Liu Y, Beyer A, Aebersold R. On the Dependency of Cellular Protein Levels on mRNA Abundance. Cell. 2016;165(3):535–50. 10.1016/j.cell.2016.03.014. 27104977 10.1016/j.cell.2016.03.014

[45] Tadej M, Mladen S, Vasilka M, Jaka H, Martin K, Gregor K, Štefan F, Uroš P. Engineering central metabolism in *Yarrowia lipolytica* increases lipid accumulation. Biochem Eng J. 2025;215:109589. 10.1016/j.bej.2024.109589.

[46] Zhu L, Zhang J, Yang J, Jiang Y, Yang S. Strategies for optimizing acetyl-CoA formation from glucose in bacteria. Trends Biotechnololgy. 2022b;40(2):149–65. 10.1016/j.tibtech.2021.04.004. 10.1016/j.tibtech.2021.04.004 33965247

[47] Bagchi SK, Patnaik R, Rawat I, Bux F. Innovative strategies for augmenting Omega-3-Fatty acid production in microalgae: Sustainable approaches for vegan food applications. Bioresour Technol. 2025;437:133176. 10.1016/j.biortech.2025.133176. 40840803 10.1016/j.biortech.2025.133176

[48] Shang C, Zhu S, Wang Z, Qin L, Alam MA, Xie J, Yuan Z. Proteome response of Dunaliella parva induced by nitrogen limitation. Algal Res. 2017;23:196–202. 10.1016/j.algal.2017.01.016.

[49] Hockin NL, Mock T, Mulholland F, Kopriva S, Malin G. The response of diatom central carbon metabolism to nitrogen starvation is different from that of green algae and higher plants. Plant Physiol. 2012;158(1):299–312. 10.1104/pp.111.184333. 22065419 10.1104/pp.111.184333 PMC3252072

[50] Kusnadi EP, Timpone C, Topisirovic I, Larsson O, Furic L. Regulation of gene expression via translational buffering. Biochim Et Biophys Acta-molecular Cell Res. 2022;1869(1). 10.1016/j.bbamcr.2021.119140. 10.1016/j.bbamcr.2021.119140 34599983

[51] Dilawar A, Ma Z, Horrocks J, Aric N, Rogers. Stress-induced Eukaryotic Translational Regulatory Mechanisms. J Clin Med Sci. 2024;8(2):1000277. 10.48550/arXiv.2405.01664. 39364184 PMC11448810

[52] Venkataramanan L, Min S, Hou SW, Jones MT, Ralston KH, Lee, Terry P. Complex and extensive post-transcriptional regulation revealed by integrative proteomic and transcriptomic analysis of metabolite stress response. Clostridium acetobutylicum Biotechnol Biofuels Bioprod. 2015;8:81. 10.1186/s13068-015-0260-9. 10.1186/s13068-015-0260-9 PMC4533764 26269711

[53] Miyakoshi M, Chao Y, Vogel J. Cross talk between ABC transporter mRNAs via a target mRNA-derived sponge of the GcvB small RNA. EMBO J. 2015;34(11):1478–92. 10.15252/embj.201490546. 25630703 10.15252/embj.201490546 PMC4474525

[54] Rau EM, Bartosova Z, Kristiansen KA, Aasen IM, Bruheim P, Ertesvåg H. Overexpression of Two New Acyl-CoA:Diacylglycerol Acyltransferase 2-Like Acyl-CoA:Sterol Acyltransferases Enhanced Squalene Accumulation in Aurantiochytrium limacinum. Front Microbiol. 2022;13:822254. 10.3389/fmicb.2022.822254. 35145505 10.3389/fmicb.2022.822254 PMC8821962

[55] Wang S, Lan C, Wang Z, Wan W, Cui Q, Song X. PUFA-synthase-specific PPTase enhanced the polyunsaturated fatty acid biosynthesis via the polyketide synthase pathway in Aurantiochytrium. Biotechnol Biofuels. 2020;13:152. 10.1186/s13068-020-01793-x. 32874202 10.1186/s13068-020-01793-x PMC7457351

[56] Song Y, Hu Z, Xiong Z, Li S, Liu W, Tian T, Yang X. Comparative transcriptomic and lipidomic analyses indicate that cold stress enhanced the production of the long C18-C22 polyunsaturated fatty acids in Aurantiochytrium sp. Front Microbiol. 2022;13., Article 915773. 10.3389/fmicb.2022.915773. 10.3389/fmicb.2022.915773 PMC9530390 36204624

[57] Dever TE, Ivanov IP, Hinnebusch AG. Translational regulation by uORFs and start codon selection stringency. Genes Dev. 2023;37(11–12):474–89. 10.1101/gad.350752.123. 37433636 10.1101/gad.350752.123 PMC10393191

[58] Sun XM, Xu YS, Huang H. Thraustochytrid Cell Factories for Producing Lipid Compounds. Trends Biotechnol. 2021b;39(7):648–50. 10.1016/j.tibtech.2020.10.008. 33199047 10.1016/j.tibtech.2020.10.008

