# Supplementary material for: Decoding nitrogen depletion–induced lipid accumulation in Aurantiochytrium sp. YHPM1 through integrated proteomic and transcriptomic analyses
Source: BMC Microbiol. 2026 Apr 21;26:526. doi: 10.1186/s12866-026-05058-9 (PMC13234984; doi:10.1186/s12866-026-05058-9)
Supplement: Supplementary file 1 — Supplementary Material 1. [file 12866_2026_5058_MOESM1_ESM.docx]

Supplementary Table S1. Primers sequences

| Primer | Sequences（5'-3'） | | Description |
| --- | --- | --- | --- |
| actin-F | GTGCTGTACTGCGTTGCACT | Forward primer for *actin* | |
| actin-R | CGCGAGGCTAGGCACAAATG | Reverse primer for *actin* | |
| acadm-F | GTAGTTGGCAAGAGGGTAAG | Forward primer for *acadm* | |
| acadm-R | GAAGCACGAGATGGAGAAG | Reverse primer for *acadm* | |
| fox2-F | GTCCCAGTTTCCAGTCTTTC | Forward primer for *fox2* | |
| fox2-R | GCGTCGTCTCAAAGGTAAG | Reverse primer for *fox2* | |
| g6pd1-F | GCTTATTACTTACTCTTGCCATG | Forward primer for *g6pd1* | |
| g6pd1-R | TCGCCTCTTGCTTGATGTCCTTC | Reverse primer for *g6pd1* | |
| g6pd2-F | AGCTCGCTCTGAAGAATGCTTG | Forward primer for *g6pd2* | |
| g6pd2-R | GAGGCTCGAAGATGTGGTCC | Reverse primer for *g6pd2* | |


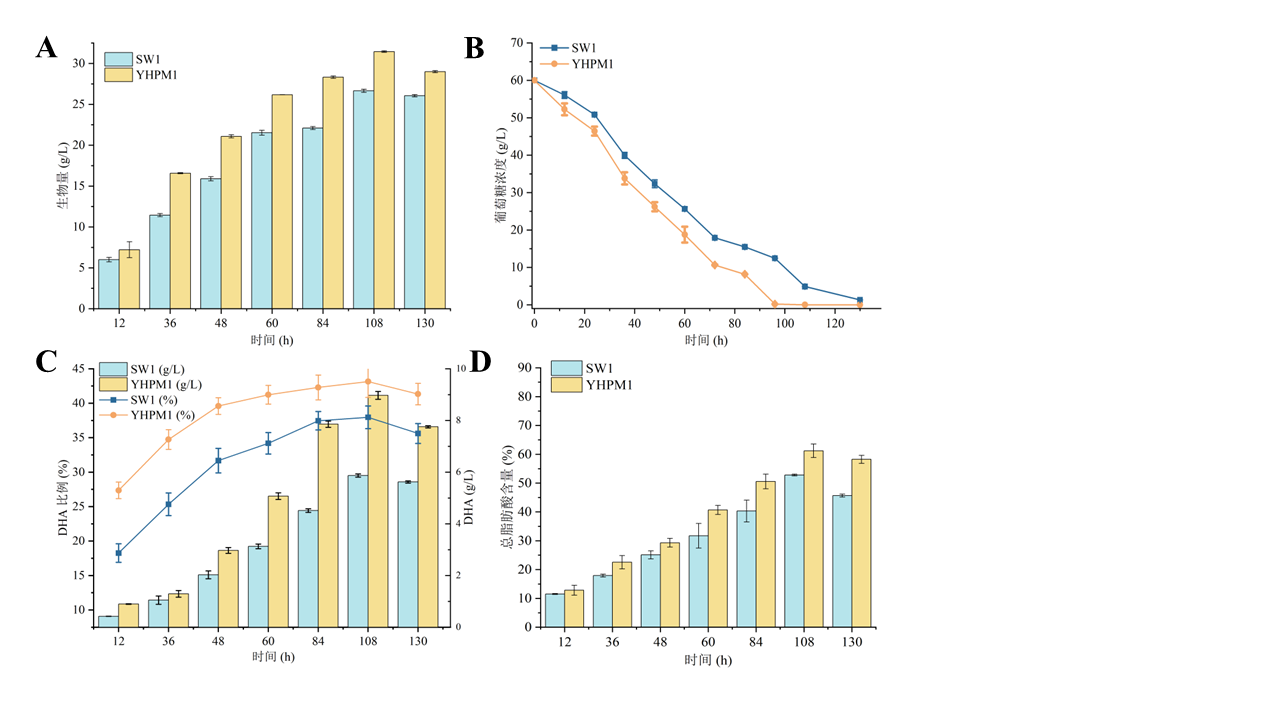


Supplementary Fig.1. Biomass and lipid production of Aurantiochytrium sp. SW1 and YHPM1. (A) Biomass; (B) Glucose concentration; (C) DHA content (%), DHA and (D) Lipid content (%).


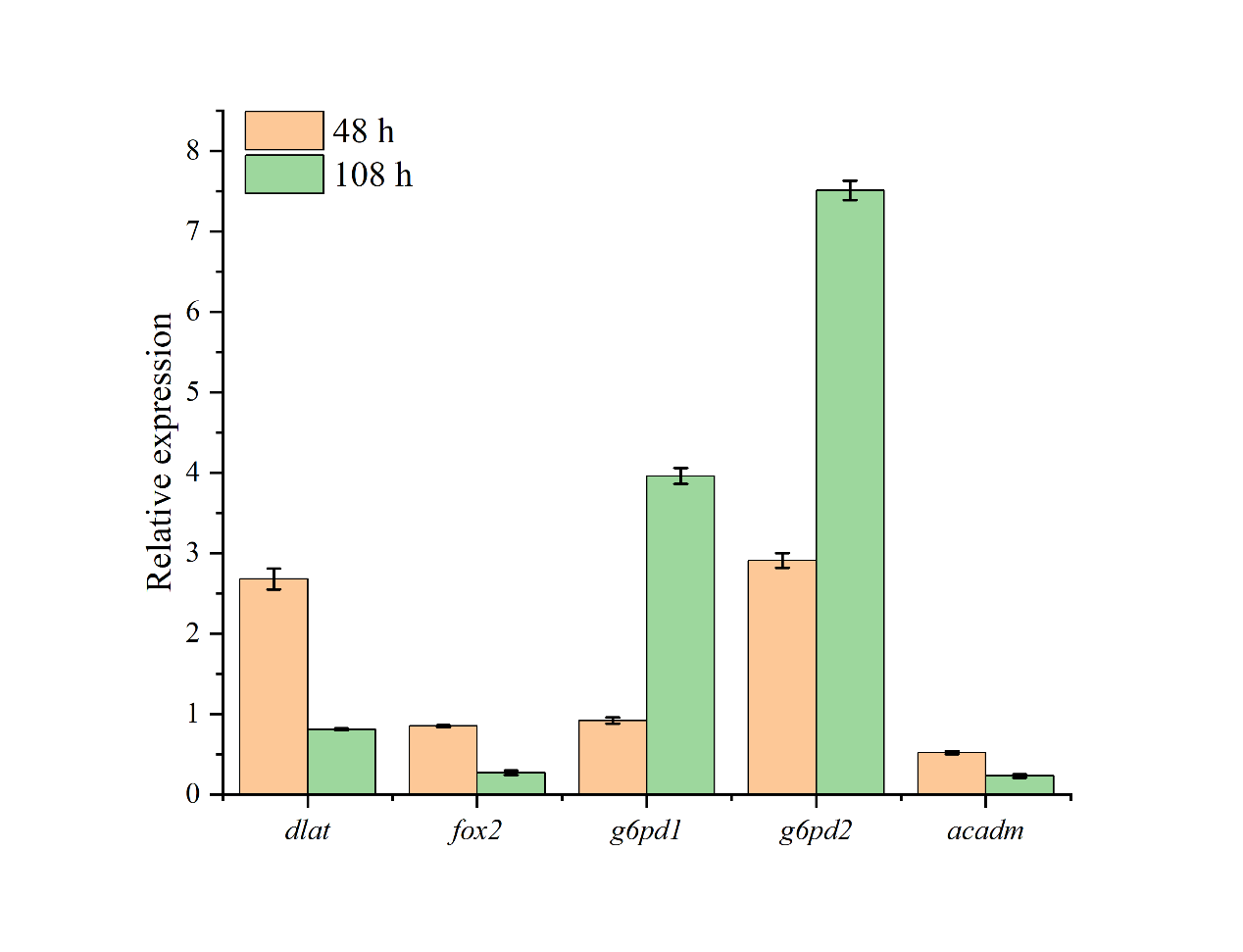


Supplementary Fig.2. Validation of gene expression by RT-qPCR
